# Distribution of *Plasmodium falciparum K*13 gene polymorphisms across transmission settings in Ghana

**DOI:** 10.1186/s12879-023-08812-w

**Published:** 2023-11-16

**Authors:** Cheikh Cambel Dieng, Victoria Morrison, Dickson Donu, Liwang Cui, Linda Amoah, Yaw Afrane, Eugenia Lo

**Affiliations:** 1 Department of Microbiology and Immunology, College of Medicine, Drexel University, Philadelphia, PA USA; 2 Department of Biological Sciences, University of North Carolina at Charlotte, Charlotte, NC USA; 3 Department of Immunology, Noguchi Memorial Institute for Medical Research, University of Ghana, Accra, Ghana; 4 Morsani College of Medicine, University of South Florida, Tampa, FL USA; 5 West Africa Center for Cell Biology of Infectious Pathogens, University of Ghana, Accra, Ghana; 6 Department of Microbiology, University of Ghana Medical School, University of Ghana, Accra, Ghana

**Keywords:** Artemisinin resistance, *Plasmodium falciparum*, Kelch13 propeller domain, Transmission zones, Codon mutation

## Abstract

Malaria is a significant global health concern, with a majority of cases in Sub-Saharan African nations. Numerous antimalarial drugs have been developed to counter the rampant prevalence of *Plasmodium falciparum* malaria. Artemisinin-based Combination Therapy (ACT) has served as the primary treatment of uncomplicated malaria in Ghana since 2005. However, a growing concern has emerged due to the escalating reports of ACT resistance, particularly in Southeast Asia, and its encroachment into Africa. Specifically, mutations in the Kelch propeller domain on chromosome 13 (*Pfk13*) have been linked to ACT resistance. Yet, our understanding of mutation prevalence in Africa remains largely uncharted. In this study, we compared *Pfk13* sequences obtained from 172 *P. falciparum* samples across three ecological and transmission zones in Ghana. We identified 27 non-synonymous mutations among these sequences, of which two of the mutations, C580Y (found in two samples from the central region) and Y493H (found in one sample from the north), had previously been validated for their association with artemisinin resistance, a phenomenon widespread in Southeast Asia. The *Pfk13* gene diversity was most pronounced in the northern savannah than the central forest and south coastal regions, where transmission rates are lower. The observed mutations were not significantly associated with geographical regions, suggesting a frequent spread of mutations across the country. The ongoing global surveillance of artemisinin resistance remains pivotal, and our findings provides insights into the potential spread of resistant parasites in West Africa. Furthermore, the identification of novel codon mutations in this study raises their potential association to ACT resistance, warranting further investigation through in vitro assays to ascertain their functional significance.

**Supplementary Information:**

The online version contains supplementary material available at 10.1186/s12879-023-08812-w.

## Introduction

Malaria is a significant global public health concern, causing around 247 million cases and 619,000 deaths worldwide [1]. More than 90% of global malaria cases are from countries in Sub-Saharan Africa where malaria is endemic [2]. Malaria transmission is determined by various factors such as environment, climate, abundance of vector mosquitoes, human migration and immune responses, and the level of malaria control measures. For example, in the northern savannah and central forest regions of Ghana, irrigation and gold mining activities create breeding grounds and/or nesting sites for *Anopheles* mosquitoes*,* contributing to high transmission rates. By contrast, in the southern coastal region with more urban setting and fewer mosquito habitats, transmission rate is relatively low [3, 4]. In addition, socioeconomic factors such as inadequate healthcare access in the rural remote areas also increase malaria incidence [4, 5]. Prior studies suggested that transmission intensity may not be necessarily associated with the prevalence of antimalarial drug resistance [6–9]. Instead, the level of host immunity [7], population size [8], and/or the number of parasite clones co-infecting the same host [9] are factors more likely affecting drug resistance.

The antimalarial drug treatment regime has undergone frequent changes over the past decades due to the rise of drug resistance. During the 1950s, Chloroquine (CQ) was the primary antimalarial drug against *P. falciparum* [10]. In 2012, WHO recommended chemoprevention with a monthly course of amodiaquine and sulfadoxine-pyrethamine (SP) to combat malaria during high transmission seasons [11]. Resistance of *P. falciparum* to CQ and SP became widespread in the 1950s and 1960s, with the first reported CQ resistance in Ghana in 1987 [12]. In response to the increased malaria related morbidity and mortality, artemisinin-based combination therapy (ACT) was recommended as the first line treatment [13]. Artemisinin has been shown to be effective against CQ sensitive and resistant *P. falciparum* malaria [14]. However, artemisinin has a short half-life and is ineffective against latent forms of primary and hepatic malaria [14]. Because artemisinin monotherapies (ART) alone did not yield unsatisfactory results, artemisinin has been combined with other antimalarial drugs that target various development stages of the parasite, particularly the early stages [15]. Selective pressure has driven to resistance against artemisinin in South America and Southeast Asia in the late 1990’s [16], and is especially prevalent in high transmission areas of Thailand and Cambodia [17]. Studies over the last decade have shown that ART resistance in *P. falciparum* is attributed to selection for mutations in genes that govern the physiological responses of the parasites and determine parasite clearance. Specifically, mutations within the highly conserved kelch propeller domain on chromosome 13 (*pfk13*) inactivate proteins needed for endocytosis of hemoglobin. Given ART is activated by the products from hemoglobin degradation, the lack of these products prevents ART activation and results in parasitic resistance [18]. *Pfk13* mutations were shown to disrupt various aspects of the parasite’s developmental program by affecting cell-cycle timing, protein response, degradation, trafficking, and mitochondrial function. Mitochondrial processes can contribute to *pfk13* mutants defense against artemisinin via temporary quiescence and rapid growth recovery, a process reversed by the mitochondrial electron transport chain inhibitor atovaquone [19]. Several codon mutations in *pfk13* gene associated with artemisinin resistance such as C580Y, Y493H, R539T, I543T, and N458Y have been identified in the field isolates from Southeast Asia and parts of Africa (Supplementary Table 1 [20, 21].

In Ghana, artemisinin combination therapy have been implemented since 2005 [3]. Though malaria transmission occurs all year round, different regions vary in transmission intensity due to climatic and landscape variations, population density, and urbanization [22]. Our previous study has shown that mutations in *Pfcrt*, *Pfmdr1*, *Pfdhfr*, and *Pfdhps* associated with CQ and SP resistance were not significantly different across the three ecological zones of Ghana, and that the majority of the isolates had the wildtype codons, with no sign of selection pressure [5]. Several non-synonymous as well as few synonymous *pfk13* mutations have been reported in the central forest region [23–25]. However, it remains unclear if the mutation frequency of *Pfk*13 differs among ecological zones. This study determined and compared *Pfk13* non-synonymous and synonymous mutations among the three ecological regions of Ghana and examined the phylogenetic relatedness between the mutant and wildtype isolates, with the goals to understand the evolution and improve the surveillance of artemisinin resistance in West Africa.

## Materials and methods

### Ethics statement

Scientific and ethical approval was given by the Institutional Scientific and Ethical Review boards of the Noguchi Memorial Institute of Medical Research, University of Ghana, Ghana and the University of North Carolina at Charlotte, USA (IRB00001276). Written informed consent/assent for study participation was obtained from all consenting parents/guardians (for minors under the age of 18), and everyone who was willing to participate in the study. All methods were reviewed and approved by the institutional review board (IRB) and performed in accordance with the relevant guidelines and regulations stated in the IRB protocols.

### Study sites and sample collection

**Fig. 1:**
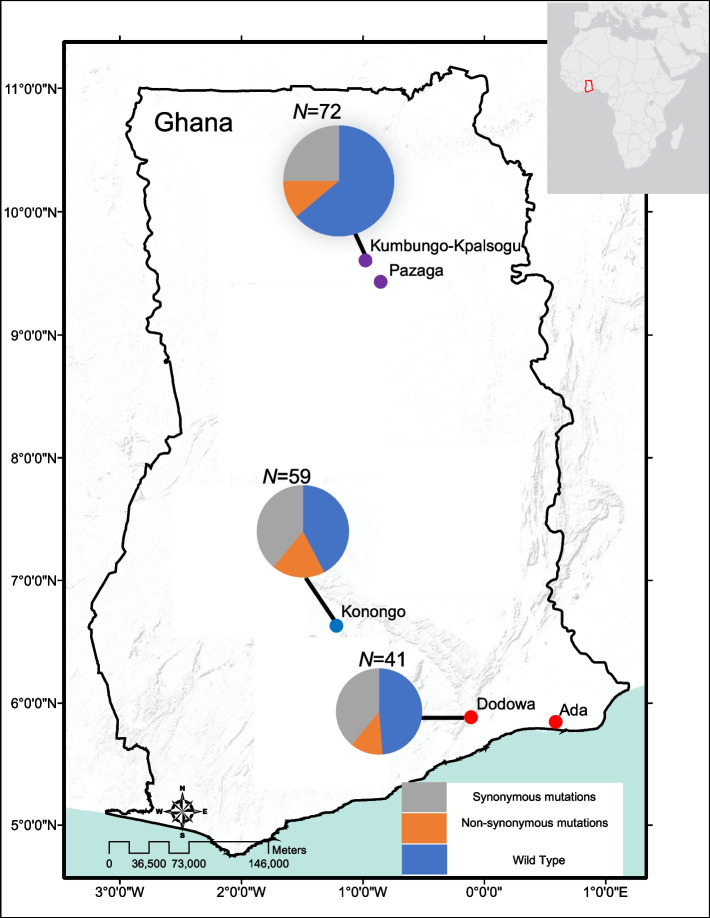
Map of Ghana showing topography and sampling. In the northern area, there were a total of *N* = 72 samples, with 64% (46) representing the wild type (WT) and 36% (25) corresponding to mutants (MT). Among the mutants, 68% were classified as synonymous, while 32% were categorized as non-synonymous. In the central region, there were *N* = 59 samples, of which 75% (44) were WT and 25% (15) were MT. Among the mutations observed, 75% were non-synonymous, whereas 25% were synonymous. Lastly, in the southern region, there were *N* = 41 samples, with 49% (20) being WT and 51% (21) being MT. Regarding the mutations in this area, 67% were synonymous, and 33% were non-synonymous. Samples with no mutations are represented in blue, samples with synonymous mutations in Grey and non-synonymous mutations in orange. The difference in size of the pie charts is proportional to the sample size

Samples were collected from five sites including Pagaza (PZ) in Tamale Municipality and Kpalsogou (KG) in Kumbungu district in the northern savannah region; Duase (KD) in Konongo in the central forest region; and Ada (AD) and Dodowa (DO) in the southern coastal region of Ghana during June–July of 2018 with a malaria prevalence of 21.4%, 24% and 14% respectively [5]. In the northern savannah zone, malaria transmission is highly seasonal and intense during/after the rainy season (June–October), whereas in the south coastal (low elevation) and central forest areas, malaria transmission is perennial [26] (Fig. 1). Finger-prick blood samples were collected from 172 asymptomatic children, aged between 3 and 13 years, who were not experiencing fever, and who attended primary schools at the designated research locations, were selected for participation. All children within this specified age range, irrespective of gender and socioeconomic background, were qualified for inclusion. However, only those whose parent or guardian granted written parental consent and obtained the child’s agreement were enrolled in the study. Thus, these individuals did not receive any antimalarial treatment prior to blood sample collection. Thick and thin blood smears were prepared for microscopic examination. Blood samples (30–50 μL) were blotted on Whatman 3MM filter papers. The filter papers were air-dried and stored in sealed plastic bags with silica gel absorbent at room temperature until DNA extraction.

Parasitic DNA was extracted from the dried blood spots using a modified version of the Chelex®-saponin method [27]. Each filter paper punch was incubated overnight at 4 °C in 1 mL of 0.5% saponin in phosphate buffered saline (PBS). The punches were washed for 30 minutes in PBS at 4 °C, transferred into new tubes containing 300 μL of 20% Chelex® and vortexed for 30 seconds. Then the tubes were heated at 95 °C for 12 minutes to elute the DNA, vortexed, and centrifuged at 10,000×*g* for 8 minutes. The supernatants (± 200 μL) were transferred into new tubes. The DNA extract was kept at 4 °C for use within a few hours or at − 20 °C for long time storage.

The SYBR Green quantitative real-time PCR (qPCR) assay of the 18S rRNA was conducted to screen for *P. falciparum* using the published protocols [28]. For the *P. falciparum-*confirmed samples, a 849-bp amplicon of the *Pfk13* gene was amplified using the published primers (Forward: GTAAAGTGAAGCCTTGTTG-3′; Reverse: 5′-TTCATTTGTATCTGGTGAAAAG − 3′) spanning nucleotide positions 1139–1979 [29]. PCR was conducted in a 20ul reaction mixture containing 2ul of genomic DNA, 10ul of 2xDreamTaq Green PCR Master Mix (Thermo Fisher) and 0.5uM primer. Reaction was performed with an initial denaturation at 94 °C for 2 min, followed by 30 cycles at 94 °C for 30 sec, 55 °C for 30 sec, and 72 °C for 60 sec, with a final 5 min extension at 72 °C. PCR products were purified prior to Sanger sequencing.

### *Pfk13* sequence analyses

We assessed the quality of Sanger sequencing chromatograms, focusing on well-resolved peaks and minimal noise using Chromas (version 2.6.6) [30] . To validate the detected mutations, we employed bioinformatics software, specifically using Clustal_W (Clustal W 2.1) [31] and BioEdit (version 7.2.5) [32], for precise sequence data analysis. This involves aligning the obtained sequences with the PF3D7 reference sequence (PF3D7_1343700) to identify the k13 mutation accurately. Confirmation is achieved through replicate sequencing reactions, and the results were rigorously evaluated and only samples that exhibited consistency in the identification of mutations were included, providing confidence in the detected genetic changes. We specifically focused on previously identified *Pfk13* mutations that have been validated for artemisinin resistance in vitro [23], in addition to other synonymous and non-synonymous mutations (Supplementary Table 2). To determine and compare sequence polymorphisms among regions, haplotype diversity (Hd; the probability that two randomly sampled alleles are different), nucleotide diversity (*Pi*; the average number of nucleotide differences per site in pairwise comparisons), and Tajima’s D value were estimated using DNASP [33]. The Tajima’s D and Fu’s FS tests were conducted to distinguish between evolving neutrally and one evolving under a non-random process, including directional or balancing selection. To infer the genetic relatedness among the *Pfk13* sequences from different transmission zones, we employed the maximum likelihood method using the Randomized Accelerated Maximum Likelihood (RAxML) for phylogenetic inferences with 500 iterations of bootstrapping for assessing confidence [34]. The resulted tree was visualized in FigTree v1.4.2 [35]. The sequence data generated in this study are available in the GenBank repository (accession number OQ102653-OQ102768).

## Results

### *Pfk13* mutations

**Fig. 2:**
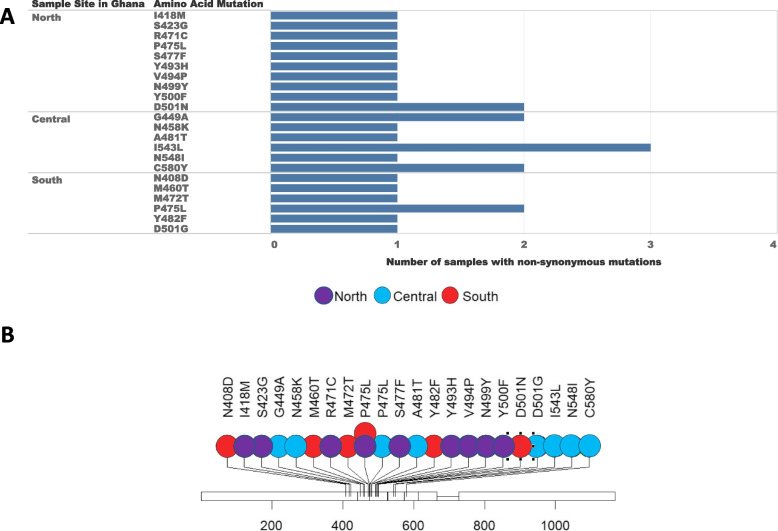
**A** Chart of the number of samples with non-synonymous mutations. In the northern region, non-synonymous mutations were identified in 15% of the samples (11 out of 72 samples) at 11 distinct codon positions. Each of these mutations was present in a single sample, accounting for 1.4% of the northern samples, except for D501N, which was observed in two samples, constituting 2.8% of the samples. In the central region, non-synonymous mutations were detected in 17% of the samples (10 out of 59 samples) at six codon positions. Mutations G449A and C580Y were each found in two samples, making up 3.4% of the samples in the central region. Mutation I543L was identified in three samples, accounting for 5% of the samples. The remaining mutations were each present in a single sample, representing 1.7% of the samples. In the southern region, non-synonymous mutations were discovered in 17% of the samples (7 out of 41 samples) at six codon positions. Mutation D501G was found in 2.4% of the samples. Furthermore, P475L was identified in two samples, constituting 5% of the samples in the south. **B** Structure of the kelch13 propeller domain, showing the position of mutations found in this study

A total of 172 *Pfk13* sequences (north = 72; central = 59; south = 41) were analysed. In the north (PZ and KG), 11 out of the 72 samples (15%) were detected with non-synonymous mutations at 11 codon positions including I418M, S423G, R471C, P475L, S477F, Y493H, V494P, N499Y, Y500F, D501N (Fig. 2). Each of these mutations was found in a single sample except D501N found in two samples. Among all codons found in the north, only Y493H has been validated in vitro that confers to ART resistance [36] (Table 1).

**Table 1:** Codon mutations distribution and function based on literature in north, central, and south Ghana. C580Y is the most prevalent non-synonymous mutation in Southeast Asia that mediates ART resistance both in vitro and in vivo. Y493H has also been validated in vitro and confers to ART resistance

Region	Codon mutation	Validation for ART resistance	Occurrence in other malaria endemic areas
**North**	I418M	No	Yes
	S423G	No	No
	P475L	No	No
	R471C	No	No
	S477F	No	Yes
	Y493H	Yes	Yes
	V494P	No	No
	N499Y	No	No
	Y500F	No	Yes
	D501N	No	No
**Central**	G449A	No	No
	N458K	No	No
	A481T	No	No
	I543L	No	No
	N548I	No	No
	C580Y	Yes	No
**South**	N408D	No	No
	M460T	No	No
	M472T	No	No
	P475L	No	No
	Y482F	No	No
	D501G	No	Yes

In the central region (KD), 10 out of the 59 samples (17%) were detected with non-synonymous mutations at six codon positions including G449A, N458K, A481T, I543L, N548I, and C580Y. Mutations G449A and C580Y were each found in two different samples; and mutation I543L was detected in three (Fig. 2). C580Y is the most prevalent non-synonymous mutation in Southeast Asia that mediates ART resistance both in vitro and in vivo [20, 36]. Other mutations including A481T, N458K, N548I were found only in a single sample, respectively. In the south (AD and DO), 7 of the 41 samples (17%) were detected with non-synonymous mutations at six codon positions including N408D, M460T, M472T, Y482F, P475L, and D501G (Fig. 2). None of these mutations has been shown to be linked to ART resistance based on prior studies (Table 1). Each of these mutations was found in a single sample, except for P475L that was also detected in a sample from the north.

Apart from non-synonymous mutations, several synonymous mutations were also found in the *Pfk13* gene among samples. In the north, there were 46 non-wildtype samples, of which 68% of the mutations were synonymous and 32% were non-synonymous. Similar proportion was observed in the south where 67% of the mutations were synonymous and 33% were non-synonymous among 21 non-wildtype samples (Fig. 1). By contrast, in the central region, 75% of the mutations were non-synonymous and 25% were synonymous among the 15 non-wildtype samples (Fig. 1).

All mutations previously reported in Ghana are compiled in Supplementary Table 3.

### Phylogenetic and diversity analyses

**Fig. 3:**
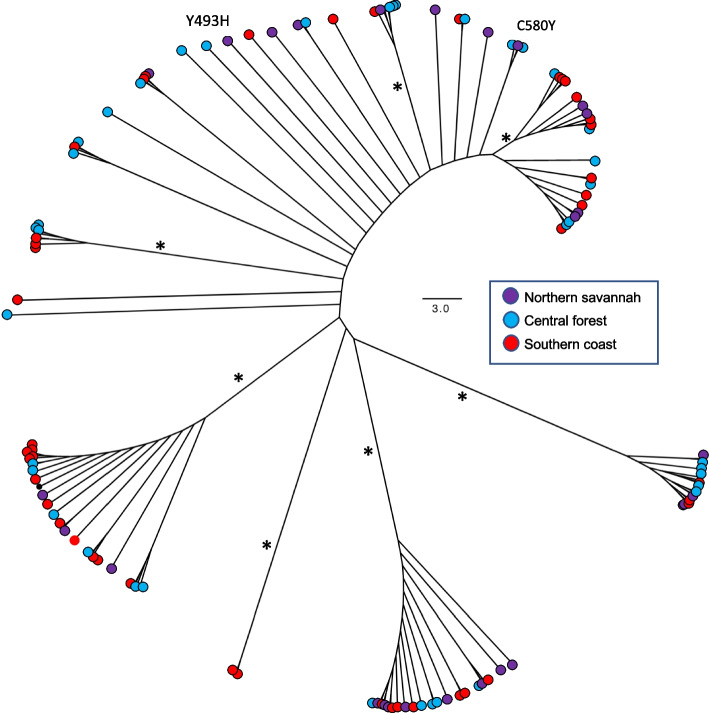
Phylogenetic tree of the 172 sequences from north central and south of Ghana. There was no clear distinction between samples from the north, central, or south as they were found in all clades. Samples containing the C580Y and Y493H both validated ART resistance mutation are highlighted. Asterisks denote clades with bootstrap values over 95%

There was no clear distinction between samples from the north, central, or south as they were found in all well-supported clades (Fig. 3). A total of 28 different *pfk*13 haplotypes were detected among the samples. Samples containing the 580Y and 493H mutations showed only one amino acid change (Supplementary Table 4). Both haplotype and nucleotide diversity were higher in the north (Hd = 0.8; Pi = 0.003) and central regions (Hd = 0.9; Pi = 0.004) than the southern region (Hd = 0.2; Pi = 0.0012; Table 2). Negative Tajima’s D values were observed in the three regions, suggesting that the Pfk13 gene is under selection pressure. The results of Fu’s FS test statistics were negative for all regions, indicating that the presence of a higher number of rare/unique Pfk13 haplotypes than what would be expected under neutrality (*p*-value = 0.005; Table 2).

**Table 2:** Haplotype diversity (Hd), nucleotide diversity (Pi), and neutrality test statistics of PfK13 gene among samples in north, central, and south Ghana. Both haplotype and nucleotide diversity were higher in the north (Hd = 0.8; Pi = 0.003) and central regions (Hd = 0.9; *Pi* = 0.004) than the southern region (Hd = 0.2; *Pi* = 0.0012). Negative Tajima’s D values were observed in the three regions, suggesting that the *Pfk13* gene is under selection pressure. The results of Fu’s FS test statistics were negative for all regions, indicating that the presence of a higher number of rare/unique *Pfk13* haplotypes than what would be expected under neutrality

Ghana	*N*	*Pi*	Hd	Hap	Neutrality test		
					Fu&Li D	Fu&Li F	Tajima D
**North**	72	0.003	0.8	55	−3.4	−2.3	−0.3
**Central**	59	0.004	0.9	47	−1.3	−1.9	−1.9
**South**	41	0.001	0.2	6	−3.4	−3.5	−2.1

## Discussion

Considering the trends of growing ART resistance in Southeast Asia and evidence of ART resistance in Sub-Saharan Africa [37], monitoring the spread of *P. falciparum* malaria and identification of *Pfk13* mutations is crucial to controlling the disease. This study highlighted a relatively high occurrence of *Pfk13* mutations in the northern and central regions of Ghana where malaria is most prevalent, compared to the southern region where transmission is low. The lack of statistical significance could likely be attributed to limited sample size. There are multiple biomarkers of ART resistance in Southeast Asia and Africa. In Southeast Asia, isolates with mutations C580Y, Y493H, R539T, I543T, and N458Y were showed with delayed clearance [20]. Of the 27 non-synonymous mutations identified in the Ghanaian samples, C580Y and Y493H has been previously validated for ART resistance and are widespread across Southeast Asia. A recent study, with a significantly larger sample size, identified 78 novel mutations that were exclusively observed in Ghana [38]. Out of these mutations, our study found only one, N458, in common. Other studies conducted between 2007 and 2016, indetified persisting mutations such as N599Y, K607E, and V637G, highlighting the ongoing need for vigilance in monitoring and responding to drug resistance [23]. However, none of these mutations have been validated for ART resistance.

The higher prevalence of *pfk*13 mutations in the north of Ghana could be attributed to a combination of factors, including the region’s poverty rate, limited access to healthcare, and a predominantly young population composed of children under 14. These factors can contribute to high malaria transmission, leading to increased genetic recombination, mutations, and spread of ART-resistant strains. The presence of shared mutations across different ecological zones suggests the role of human migrations and parasite gene flow across the country, emphasizing the intricate interplay among transmission dynamics, human movement, and parasite evolution in the context of artemisinin resistance [39]. In the north, malaria transmission is seasonal and significantly higher during and after the rainy season. Consistent to other studies, parasites from the southern region have low prevalence of *Pfk13* mutations [26]. Despite the humid and warmer weather near the coast climate that is optimal for mosquito development, the southern region is densely populated, highly urbanized, higher socio-economic status, and greater access to healthcare services [40]. These factors likely contribute to low incidence of malaria, as people living in this area are more likely to be diagnosed and treated promptly for malaria.

The central forest region serves as a transitional zone between the north savannah and urban south; and reflects how the parasitic prevalence and variability may change as urbanization occurs. The central region of Ghana has the country’s major mineral reserves. Large-scale mining activity leads to landscape damage and pockets of stagnant water favor mosquito development [41]. In this region, we found relatively low frequency of *Pfk13* mutations. One of the mutations G449A was previously identified as a candidate mutation for ART surveillance in Southeast Asia [20]. The type of amino acid substitution is also important when inferring the impact on resistance. For example, while I543L has not been yet confirmed as a mutation resulting in ART resistance, I543T was validated as a key marker for ART resistance [20]. Further analysis by CCF53_62 matrices that determines the effect of amino acid changes to protein function could offer deeper insights into the significance of the type of substitutions [42]. Other codon mutations associated with delayed ART clearance among African *P. falciparum* parasites include Q613H reported in Senegal [43], P574L in Rwanda [44] and Henan Province of China [45]), A675V in Rwanda [44] and Uganda [46], P553L in Kenya [47], and K189T in Senegal [43] and Uganda [48]. Previous study has shown that the mutation 580Y was observed in Chinese migrant workers returning from Ghana [49]. A study conducted between 2007 and 2016 in Ghana have reported a high number of mutations of which 77% was nonsynonymous [23], though their functional significance still remains unclear and warrants further investigations. Though not validated in function, A578S is the most commonly reported non-synonymous mutation in different parts of Africa including Ghana [50, 51] Equatorial Guinea [52], Mali [53], Kenya [54], Togo [55], but this mutation was not found in this study.

Based on neutrality tests, the *Pfk13* gene was detected with significance selection pressure, particularly in the central region where there is a greater ratio of nonsynonymous to synonymous substitutions. It is plausible that easy access to antimalarial drugs in this region accelerates the development of selection pressure to ART resistance. With more than 10,000 licensed chemical seller (LCS) shops in Ghana, these shops are often the first point of care for febrile illnesses such as malaria, particularly in hard-to-reach areas [56]. Due to the increased accessibility of antimalarials, patients tend to opt for self-treatment, which can result in inadequate and inappropriate usage of non-prescribed drugs [56, 57]. As there are more LCS shops relative to health facilities in rural areas, artemisinin combination therapy could become more available in the northern savannah region and increase the *Pfk13* mutation frequency. Many mutant *Pfk13* codons in Ghana were found to be zone specific, implying that intra-country gene flow could be limited in spreading parasites through human migration [23]. While Sanger sequencing remains a valuable tool, its sensitivity constraints often hinder the accurate detection of minor mutants. Amplicon deep sequencing holds promises for unveiling a more comprehensive and nuanced mutational landscape, particularly in complex scenarios like polyclonal and mixed infections. Another limitation of this study is the lack of clinical efficacy data. Therefore, novel candidate mutations should be examined for both in vivo and in vitro resistance to artemisinin to clarify the significance of the mutations. While the *Pfk13* gene is a valuable marker for understanding artemisinin resistance and genetic diversity, it might not reveal a full picture of the resistance mechanisms. Whole-genome sequencing and analysis of additional genetic markers may offer more comprehensive insights into the overall genetic diversity, evolution, and potential resistance mechanisms of *P. falciparum*.

## Conclusion

The global surveillance of artemisinin resistance is and will continue to be crucial to understanding how antimalarial drug resistance evolves and spreads. This study highlighted a relatively high occurrence of *Pfk13* mutations in the northern and central regions of Ghana where malaria is most prevalent, compared to the southern region where transmission is low. Our sequence analyses revealed 27 non-synonymous mutations and most of them have not been validated for ART resistance nor previously reported elsewhere in Africa. Our findings emphasize the need for further clinical and/or in vitro testing of the functional significance of novel *Pfk13* codon mutations. Future studies should investigate the association of transmission intensity with *pfk13* mutation prevalence at a broader geographical scale. Understanding how factors such as intra- and inter-country migration patterns and urbanization affect parasitic diversity and antimalarial resistance will have great implications on controlling malaria spread in Africa.

### Supplementary Information

**Additional file 1.**

## Data Availability

The datasets generated and/or analyzed during the current study are available in the GenBank repository, [GenBank accession number BankIt2656762: OQ102653 - OQ102768.]
