# Supplementary material for: Distribution of Plasmodium falciparum K13 gene polymorphisms across transmission settings in Ghana
Source: BMC Infect Dis. 2023 Nov 16;23:801. doi: 10.1186/s12879-023-08812-w (PMC10652499; doi:10.1186/s12879-023-08812-w)
Supplement: Supplementary file 1 — Additional file 1. [file 12879_2023_8812_MOESM1_ESM.docx]

**Supplemental Table 1.** Candidate K13 artemisinin resistance codon mutations. A578S is the most reported non-synonymous mutation in different parts of Africa.

| Mutation |  |
| --- | --- |
| T474I  T508N  P527T  G533S  N537I  S623C | Candidate Arteminsin resistance K13 propeller mutations in SEA[1] |
| C469C  R561H  A578S  M579I  M608l  E612D  Q613L  N629Y | Candidate Arteminsin resistance K13 propeller mutations in Africa[2] |

**Supplemental Table 2**. Validated K13 artemisinin resistance codon mutations and non-validated non-synonymous mutations in Southeast Asia (SEA) and Africa.

| Mutation |  |
| --- | --- |
| C580Y  R539T  I543T  Y493H  N458Y  M476I  N537D  G538V  P667T  C469F  F673I  E252Q  G449A  C469Y  A481V  V568G  D584V  E556D  R575K  S621F  D353Y  P441L | Validated Arteminsin resistance K13 propeller mutations in SEA[1, 3]  Non-validated non-synonymous K13 mutations in SEA[1, 3] |
| R539T  C580Y  Y493H  I543T | Validated Arteminsin resistance K13 propeller mutations in Africa |

**Supplemental Table 3.** All reported K13 artemisinin resistance codon mutations in Ghana.

| **Mutations** | **Region** | **Ref** |
| --- | --- | --- |
| N408S | Forest (Begoro, Bekwai, Hohoe, Sunyami, Tarkwa), Ghana | [4] |
| L422F | Forest (Begoro, Bekwai, Hohoe, Sunyami, Tarkwa), Ghana |  |
| S466N | Forest (Begoro, Bekwai, Hohoe, Sunyami, Tarkwa), Ghana |  |
| R515I | Forest (Begoro, Bekwai, Hohoe, Sunyami, Tarkwa), Ghana |  |
| R539I | Forest (Begoro, Bekwai, Hohoe, Sunyami, Tarkwa), Ghana |  |
| C542W | Forest (Begoro, Bekwai, Hohoe, Sunyami, Tarkwa), Ghana |  |
| E606D | Forest (Begoro, Bekwai, Hohoe, Sunyami, Tarkwa), Ghana |  |
| K607E | Forest (Begoro, Bekwai, Hohoe, Sunyami, Tarkwa), Ghana |  |
| W470E | Cape Coast, Central Region, Ghana | [5] |
| S549T | Cape Coast, Central Region, Ghana |  |
| S550Y | Cape Coast, Central Region, Ghana |  |
| Q613H | Cape Coast, Central Region, Ghana |  |
| G690V | Cape Coast, Central Region, Ghana |  |
| Y482H | Cape Coast, Central Region, Ghana |  |
| S522C | Cape Coast, Central Region, Ghana |  |
| V566I | Cape Coast, Central Region, Ghana |  |
| E455G | Accra, Greater Accra, Ghana | [6] |
| N458D | Accra, Greater Accra, Ghana |  |
| A481C | Accra, Greater Accra, Ghana |  |
| T535A | Accra, Greater Accra, Ghana |  |
| Y616S | Accra, Greater Accra, Ghana |  |
| L618V | Accra, Greater Accra, Ghana |  |
| A621G | Accra, Greater Accra, Ghana |  |
| L663I | Accra, Greater Accra, Ghana |  |
| N672I | Accra, Greater Accra, Ghana |  |
| K479N | Fanteakwa, Eastern Region, Ghana | [5] |
| V494I | Fanteakwa, Eastern Region, Ghana |  |
| G690V | Fanteakwa, Eastern Region, Ghana |  |
| M460I | Fanteakwa, Eastern Region, Ghana |  |
| E461D | Fanteakwa, Eastern Region, Ghana |  |
| I540S | Fanteakwa, Eastern Region, Ghana |  |
| S550Y | Fanteakwa, Eastern Region, Ghana |  |
| A676S | Fanteakwa, Eastern Region, Ghana |  |

**Supplemental Table 4.** Hplotypes of K13 propeller observed in Ghana. Sequence alignment of the 28 *P. falciparum* haplotypes showing polymorphisms in the K13 propeller gene.

| **Sample** | **Region** | **N408** | **I418** | **S423** | **G449** | **N458** | **M460** | **R471** | **M472** | **P475** | **S477** | **F491** | **Y493** | **V494** | **N499** | **Y500** | **D501** | **I543** | **N548** | **C580** |
| --- | --- | --- | --- | --- | --- | --- | --- | --- | --- | --- | --- | --- | --- | --- | --- | --- | --- | --- | --- | --- |
| 3D7 | N/A | **N** | **I** | **S** | **G** | **N** | **M** | **R** | **M** | **P** | **S** | **F** | **Y** | **V** | **N** | **Y** | **D** | **I** | **N** | **C** |
| PZ032 | North |  |  |  |  |  |  |  |  |  |  |  |  |  |  |  |  |  |  |  |
| PZ092 | North |  | **M** | **G** |  |  |  |  |  |  |  |  |  |  |  |  |  |  |  |  |
| PZ103 | North |  |  |  |  |  |  |  |  |  | **F** |  |  |  |  |  |  |  |  |  |
| PZ107 | North |  |  |  |  |  |  |  |  |  |  |  | **H** |  |  |  |  |  |  |  |
| PZ126 | North |  |  |  |  |  |  |  |  |  |  |  |  |  |  |  | **N** |  |  |  |
| PZ128 | North |  |  |  |  |  |  |  |  |  |  |  |  |  |  |  |  |  |  |  |
| PZ132 | North |  |  |  |  |  |  | **C** |  |  |  |  |  |  | **Y** |  |  |  |  |  |
| PZ133 | North |  |  |  |  |  |  |  |  |  |  |  |  |  |  |  |  |  |  |  |
| PZ134 | North |  |  |  |  |  |  |  |  |  |  |  |  | **P** |  |  |  |  |  |  |
| PZ136 | North |  |  |  |  |  |  |  |  |  |  |  |  |  |  | **F** |  |  |  |  |
| PZ141 | North |  |  |  |  |  |  |  |  |  |  |  |  |  |  |  | **H** |  |  |  |
| PZ142 | North |  |  |  |  |  |  |  |  | **L** |  |  |  |  |  |  |  |  |  |  |
| KD010 | Central |  |  |  | **A** |  |  |  |  |  |  |  |  |  |  |  |  |  |  |  |
| KD015 | Central |  |  |  |  |  |  |  |  |  |  |  |  |  |  |  |  |  |  | **Y** |
| KD018 | Central |  |  |  |  |  |  |  |  |  |  |  |  |  |  |  |  | **L** |  |  |
| KD025 | Central |  |  |  |  |  |  |  |  |  |  |  |  |  |  |  |  | **L** |  |  |
| KD028 | Central |  |  |  |  | **K** |  |  |  |  |  |  |  |  |  |  |  |  | **I** |  |
| KD045 | Central |  |  |  |  |  |  |  |  |  |  |  |  |  |  |  |  |  |  | **Y** |
| KD159 | Central |  |  |  |  |  |  |  |  |  |  |  |  |  |  |  |  | **L** |  |  |
| KD172 | Central |  |  |  | **A** |  |  |  |  |  |  |  |  |  |  |  |  |  |  |  |
| ADA045 | South |  |  |  |  |  |  |  |  | **L** |  |  |  |  |  |  |  |  |  |  |
| ADA079 | South |  |  |  |  |  |  |  | **T** |  |  |  |  |  |  |  |  |  |  |  |
| ADA105 | South |  |  |  |  |  |  |  |  | **L** |  |  |  |  |  |  |  |  |  |  |
| ADA106 | South |  |  |  |  |  |  |  |  |  |  |  |  |  |  |  |  |  |  |  |
| ADA109 | South |  |  |  |  |  |  |  |  |  |  |  |  |  |  |  |  |  |  |  |
| ADA123 | South | D |  |  |  |  |  |  |  |  |  |  |  |  |  |  |  |  |  |  |
| ADA150 | South |  |  |  |  |  |  |  |  |  |  |  |  |  |  |  | **G** |  |  |  |
| ADA153 | South |  |  |  |  |  | **T** |  |  |  |  |  |  |  |  |  |  |  |  |  |

**References**

1. Ashley, E.A., et al., *Spread of artemisinin resistance in Plasmodium falciparum malaria.* New England Journal of Medicine, 2014. **371**(5): p. 411-423.

2. Stokes, B.H., et al., *Plasmodium falciparum K13 mutations in Africa and Asia impact artemisinin resistance and parasite fitness.* Elife, 2021. **10**.

3. Fairhurst, R.M. and A.M. Dondorp, *Artemisinin-Resistant Plasmodium falciparum Malaria.* Microbiol Spectr, 2016. **4**(3).

4. Matrevi, S.A., et al., *Plasmodium falciparum kelch propeller polymorphisms in clinical isolates from Ghana from 2007 to 2016.* Antimicrobial agents and chemotherapy, 2019. **63**(11): p. e00802-19.

5. Mensah, B.A., et al., *Antimalarial Drug Resistance Profiling of Plasmodium falciparum Infections in Ghana Using Molecular Inversion Probes and Next-Generation Sequencing.* Antimicrob Agents Chemother, 2020. **64**(4).

6. Tornyigah, B., et al., *Effect of Drug Pressure on Promoting the Emergence of Antimalarial-Resistant Parasites among Pregnant Women in Ghana.* Antimicrob Agents Chemother, 2020. **64**(6).
